# Influence of coronary flow profiles on bolus shape and quantitative myocardial perfusion MRI

**DOI:** 10.1186/1532-429X-14-S1-W73

**Published:** 2012-02-01

**Authors:** Dirk Graafen, Julia Hamer, Regine Schmidt, Melanie Schmitt, Stefan Weber, Laura M Schreiber

**Affiliations:** 1 Mainz University Medical Center, Mainz, Germany; 2 Max-Planck-Graduate Center with the Johannes Gutenberg-University, Mainz, Germany

## Summary

Simulations of the influence of contrast agent bolus dispersion on the shape of the contrast agent bolus and on quantification of myocardial blood flow (MBF) have been made using an exponential residue function model, and an computational fluid dynamics (CFD) approach. Based on the exponential residue function model, up to 20% underestimation of MBF appeared reasonable. CFD simulations showed that bolus dispersion occures in normal coronary vessels as well as in those with stenosis. However, bolus dispersion was smaller with stenosis than without.

## Background

Measurement of myocardial flood flow relies on an estimation of contrast agent inflow into the particular tissue of interest, i.e. the arterial input function (AIF). The AIF is usually measured in the left ventricular cavity. However, due to laminar or more complex flow profiles, bolus dispersion may occur in the coronary artery before the bolus reaches the myocardium. This additional bolus duration would then misleadingly be attributed to dispersion in the tissue of interest, i.e. a longer duration of the tissue residue function. As a consequence, an underestimation of MBF would result. However, it was unclear if these effects occur.

Since a direct measurement of bolus dispersion is difficult, it was the aim of this study to estimate the influence of bolus dispersion on MBF quantification.

## Methods

Simulations of the influence of contrast agent bolus dispersion on quantification of MBF have been made using a exponential residue function model (time constant 1-6s), and computational fluid dynamics (CFD). With CFD, the bolus dispersion was calculated for stationary and pulsatile blood flow, and for linear, curved vessels as well as for different degrees of stenosis.

## Results

Based on the exponential residue function model, up to 20% underestimation in MBF appears reasonable. With the CFD approach, a more detailed and realistic analysis was feasible. Contrary to our expectations, stenosis resulted in a smaller bolus dispersion than a normal vessel. Errors of MBF estimates were less than 10%. Differences in bolus dispersion between stationary and pulsatile blood flow were small.

## Conclusions

Bolus dispersion may influence MBF measurement. Unexpectedly, stenosis results in less bolus dispersion than that observed in the healthy situation. Therefore, it may be speculated that blood flow estimates in patients with coronary arteries may be more realistic than those in normals.

Although our simulations focused on quantitative MRI measurements, we assume that our results may also apply qualitatively for semiquantitative MBF estimates, i.e. the upslope technique.

## Funding

German Research Foundation SCHR 687/1.

